# Income and education level trajectories and changes in the daily consumption of vegetables after thirteen years of follow-up: the *Pró-Saúde* Study

**DOI:** 10.1590/1980-549720240043

**Published:** 2024-08-30

**Authors:** Talita Lelis Berti, Diana Barbosa Cunha, Rosely Sichieri, Joana Maia Brandão, Eduardo Faerstein

**Affiliations:** I Universidade Estadual do Rio de Janeiro, Social Medicine Institute, Department of Epidemiology, – Rio de Janeiro (RJ), Brazil.

**Keywords:** Food consumption, Vegetables, Socioeconomic factors, Social iniquity, Consumo alimentar, Vegetais, Fatores socioeconômicos, Desigualdade social

## Abstract

**Objective:**

This study aimed to examine whether education level and income trajectories influence vegetable consumption changes over 13 years among civil servants at different campuses of a university in the state of Rio de Janeiro, Brazil.

**Methods:**

Vegetable intake frequency (daily and non-daily consumption), income (per capita), and education level (maintenance of low schooling/ upward mobility/maintenance of high schooling) were assessed at baseline (1999) and in the fourth wave (2011–12) of the *Pró-Saúde* (Pro-Health) cohort study. A total of 2,381 participants were analyzed. The association between educational and income trajectories and variation in vegetable consumption was assessed via crude and age-adjusted generalized linear models, stratified by sex.

**Results:**

Men in upward educational mobility showed a 0.5% increase in vegetable consumption (p=0.01), while women in this group demonstrated a 2.5% increase (p=0.05). Adjusted models showed that women who reduced their income had a lower likelihood of consuming vegetables (odds ratio [OR] 0.93; 95% confidence interval [CI] 0.89–0.97).

**Conclusions:**

The findings highlight the influence of social inequalities on vegetable consumption in adults.

## RESUMO

**Objetivo:**

O objetivo deste estudo foi avaliar se as trajetórias do nível de educação e de renda influenciam na mudança do consumo de vegetais ao longo de 13 anos entre funcionários públicos de diferentes campi de uma universidade do Rio de Janeiro, Brasil.

**Métodos:**

A frequência de consumo de vegetais (consumo diário e não diário), a renda (*per capita*) e o nível de educação (manutenção da baixa escolaridade/mobilidade ascendente/ manutenção da alta escolaridade) foram avaliados no início (1999) e na quarta onda (2011–12) do estudo de coorte Pró-Saúde. Foram analisados 2.381 participantes, e a associação entre as trajetórias de escolaridade e renda e a variação no consumo de vegetais foi avaliada via modelos lineares generalizados brutos e ajustados por idade e variação da escolaridade e estratificados por sexo.

**Resultados:**

Homens em mobilidade educacional ascendente apresentaram aumento de 0,5% no consumo de vegetais (p=0,01), enquanto mulheres nesse grupo demonstraram aumento de 2,5% (p=0,05). Modelos ajustados mostraram que mulheres que reduziram sua renda apresentaram menor probabilidade de consumir vegetais (*odds ratio* [OR] 0,93; intervalo de confiança [IC] 95% 0,89–0,97).

**Conclusão:**

Os achados destacam a influência das desigualdades sociais no consumo de vegetais em adultos.

## INTRODUCTION

Vegetable consumption is a significant indicator of healthy eating, and low consumption is one of the modifiable risk factors for chronic non-communicable diseases (NCDs), particularly cardiovascular diseases and cancer^1–3^. Despite this, the global average consumption of fruits and vegetables falls below the World Health Organization's (WHO) recommended amount of 400 grams per day^4^.

Socioeconomic factors such as education level, income, and sex are significant determinants of vegetable consumption^5–9^. These factors can affect the availability, accessibility, and affordability of these foods, and knowledge and awareness of their health benefits^10^.

However, the independent effect of education and income trajectories on changes in food intake still needs to be explored. It is investigated predominantly in cohorts from high-income countries such as Japan^11^, Finland^12^, United Kingdom^13^, and Spain^14^. These studies indicate a direct association between remaining stable in higher occupational classes (non-manual workers) and healthier food consumption.

A detailed study on the evolution of dietary consumption over the past ten years shows that the Brazilian diet is still characterized by the consumption of traditional foods such as rice and beans, along with a high-frequency intake of ultra-processed foods like biscuits and sodas. The vegetable consumption frequency has increased slightly from 41.9 to 44.5%^15^. In developing nations such as Brazil, households with higher incomes have access to a more diverse diet that includes fruits and vegetables at a higher cost and ultra-processed foods. On the other hand, households with lower incomes are exposed to a monotonous diet of lesser monetary value^16^.

Typically, a higher education level correlates with an elevated understanding of health, thereby fostering more favorable health-related behaviors. A recent Brazilian study indicates that individuals with higher educational attainment are more inclined to consume fresh or minimally processed foods. In contrast, those with intermediate education levels show a higher frequency of ultra-processed food consumption^17^.

Brazil has undergone significant economic, social, and demographic transformations in the last two decades^18^. During this time, the illiteracy rate decreased. In 2022, it reached 5.6%, the lowest rate of the series. Furthermore, the percentage of people with higher education hiked, as did those with complete secondary education^19^. The average schooling of the population aged 25 years and older increased from 6.0 to 7.6 years of complete education in 2015. In 2022, the average schooling was 9.9 years. The proportion of people aged 25 years or over who completed at least compulsory primary education — that is, completed at least secondary education — reached 53.2% in 2022, exceeding half of the population for the first time^19,20^.

Higher education is associated with greater economic empowerment, and since the prices of vegetables are often higher than those of ultra-processed foods, they serve as strong determinants for dietary choices^21^.

Although there is some evidence of the relationship between socioeconomic differences in consumption^22^, evidence from longitudinal studies still needs to be provided. To date, we identified only three Brazilian cohort studies that investigated the association between intragenerational social mobility (exposure) and food consumption as an outcome^23–25^. However, in these studies, food consumption was assessed only in the last wave of follow-up, without investigating changes over time, and therefore, a longitudinal analysis approach was not used. Thus, our manuscript advances the investigation of the effect of schooling and income trajectories on changes in vegetable consumption over time, as no other longitudinal study with national data with the same objective has been observed to date. Understanding how income and education impact vegetable consumption, one of the fundamentals of a healthy diet is a crucial step in mitigating social inequalities in health.

Thus, the present study aimed to evaluate the effect of education and income trajectories on vegetable consumption change in a Brazilian cohort of civil servants, after 13 years of follow-up.

## METHODS

**Figure 1:**
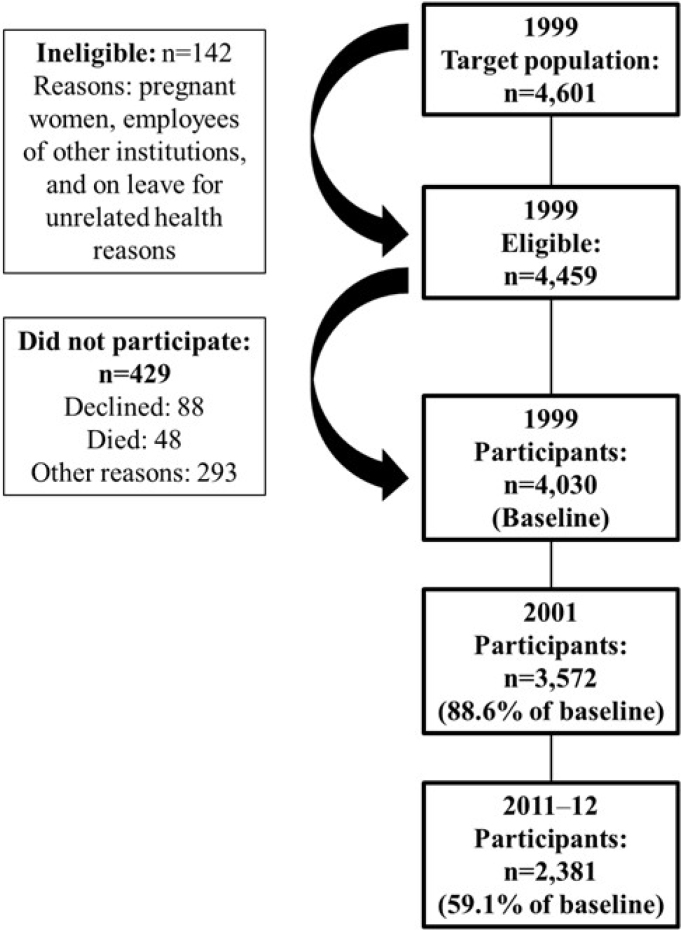
Flowchart of study participation and loss to follow-up. *Pró-Saúde* (Pro-Health) Study, Rio de Janeiro, Brazil, from 1999 to 2011–12.

Data derive from the *Pró-Saúde* (Pro-Health) Study, a prospective cohort of civil servants on a university campus of the Rio de Janeiro State University, Brazil. All technical-administrative servants, effective in 1999, and aged between 18 and 65 were invited to participate in the study's baseline (n=4,601). Three follow-up waves were conducted in 2001–2, 2006–7, and 2011–12. The present study population consisted of employees who participated at baseline and the last wave, totaling 2,381 employees over a 13-year follow-up period (59.1% of baseline participants). Figure 1 illustrates the study participation in a flowchart.

Data were collected during employees’ working hours, using a self-reported questionnaire, gathering information regarding socioeconomic and sociodemographic characteristics and food consumption (available at: http://rede-prosaude.org/). The questionnaires were revised, and data were entered twice and independently, using Epi Info software version 6.0 (Centers for Disease Control and Prevention, Atlanta, United States). A reliability analysis (test-retest) of the questionnaire was performed, reapplying in subsamples of 98 participants at baseline (0.67 to 0.79 kappa)^26^ and 58 participants in the third wave of the cohort, with good results.

Vegetable consumption was assessed in 1999 and 2011–12 by the question: "How often do you consume vegetables?"

Five response options were offered to the participants:

- never or less than once a month;
- 1 to 3 times a month;
- 1 to 3 times a week;
- 4 to 6 times a week; or
- daily.

Data were analyzed as daily and non-daily consumption. Education level was assessed within seven categories:

1. Incomplete primary education and
2. Complete;
3. Incomplete secondary education and
4. Complete;
5. Incomplete higher education and
6. Complete; and
7. Postgraduate or more. Subsequently, education level was re-categorized into three categories: up to complete primary education (including incomplete secondary education); complete secondary education (including incomplete higher education), and complete higher education or above.

Education level trajectory was obtained by comparing 1999 and 2011–12 responses.

Three categories were created:

1. Maintenance of low schooling, when the individual remained at a level of education equivalent to complete primary or secondary education;
2. Upward mobility, when there was any rise in the schooling level; and
3. Maintenance of high schooling, in the case of continuing at the level of schooling equivalent to complete higher education or any level above.

The question about household income had ten response categories in income strata:

1. Up to R$1,000;
2. Between R$1,001 and R$1,500;
3. Between R$1,501 and R$2,000;
4. Between R$2,001 and R$2,500;
5. Between R$2,501 and R$3,000;
6. Between R$3,001 and R$4,000;
7. Between R$4,001 and R$5,000;
8. Between R$5,001 and 6,000;
9. Between R$6,001 and 7,000; and
10. More than R$ 7,000.

The average value of the first income category was estimated at R$501, and that of the last category at R$9,429.16, using formulas based on the Pareto income distribution curves described by Parker & Fenwick^27^, to obtain the average value relating to these categories.

The per capita income in minimum wage (MW) was used, with the baseline income corrected for inflation, based on the Special Extended National Consumer Price Index (IPCA-E, in Portuguese) of the Brazilian Institute of Geography and Statistics (IBGE, in Portuguese), from 1992 onwards, using the Brazilian Central Bank's online calculator and considering the MW of R$622 in July 2012 (or US$428).

Tertiles of per capita MW evaluated change in income by comparing income tertiles between 1999 and 2011–12, resulting in four categories:

1. Maintenance in the first or second tertile;
2. Upward mobility;
3. Downward mobility, and
4. Maintenance in the third tertile.

For data analysis, the absolute and relative frequencies of each category of education trajectory and daily vegetable consumption were described, stratified by sex.

The odds ratio (OR) was obtained using generalized linear models via the PROC GENMOD procedure in Statistical Analysis System (SAS), with a log link function and Poisson distribution. This modeling considers the inter- and intra-individual correlations of observations over time and allows the analysis of data collected at unequal intervals between study waves, besides being the most appropriate option when individuals are lost to follow-up and missing outcome values^28^. The model was built with a binary outcome: daily vegetable intake change (reference=0, non-daily). The independent variables were income trajectory, considering maintenance in the first or second tertiles of per capita household income as a reference category, and education trajectory, considering maintenance in low education as a reference category. The age of the participants in each follow-up wave was included in the model to reflect the variation of the variables in time due to the different spacing between the study waves. The model was also stratified by sex.

Descriptive statistical analyses were performed using Stata statistical software version 13.0 (StataCorp), and longitudinal analyses were performed using SAS version 9.4 (SAS Institute Inc, Cary, NC).

The study was approved by the Research Ethics Committees of the Institute of Social Medicine of Rio de Janeiro State University and the Antônio Pedro University Hospital on May 10, 1999, December 13, 2001, and October 18, 2011 (CAAE 0041.0.259.000-11). Participation was voluntary, and all participants signed an informed consent form.

## RESULTS

The percentage of missing data was 0.5% in the variable referring to the consumption of vegetables in the two waves of the study. The percentage of missing data on the education or income trajectories was 1.1%, mainly due to the non-declaration of the number of income dependents. Participants with missing data on education or income trajectories were excluded from the study population, which then totaled 2,381 employees.

In 1999, at the study's baseline, females accounted for 56.3%, with most being young adults (mean age 39 years, ranging from 22 to 67 years) and having at least a high school level, as shown in Table 1. The mean per capita income was 2.43 times the MW, ranging from 0.20 to 20.9 times (data not shown in tables).

**Table 1:** General characteristics of the study population at the baseline of the *Pró-Saúde* (Pro-Health) Study cohort, Rio de Janeiro, Brazil, 1999.

Characteristics		n	%
Gender			
	Male	1,041	43.7
	Female	1,340	56.3
Age (years)			
	22–34	682	28.6
	35–44	1,136	47.7
	45–54	461	19.4
	55–67	102	4.3
Education level			
	Incomplete high school	521	21.9
	Complete high school or incomplete higher education	906	38.0
	Higher education or more	954	40.1
Per capita income in minimum wages (tertiles)			
	1° tertile (<1.39 MW)	832	34.9
	2° tertile (1.39≤MW≥2.8)	904	38.0
	3° tertile (>2.8 MW)	645	27.1
Total		2,381	100.0

MW: minimum wage.

Between 1999 and 2011–12, 44.1% of men had incomplete high school education and remained in that category, and 42.6% continued in the first or second income tertile. During this period, there was an overall increase of 1.1% in the daily consumption of vegetables. By 2012, 28.9% of men reported consuming vegetables daily (Table 2).

**Table 2:** Schooling and income trajectories and daily vegetable consumption according to socioeconomic trajectories among male civil servants at a university, in 1999 and 2011–12: *Pró-Saúde* (Pro-Health) Study, Rio de Janeiro, Brazil (n=1,041).

Socioeconomic trajectories		Total		Daily vegetable consumption			
				1999	2012	Δ	p-value
		n	%	%	%	%	
Schooling trajectory							
	Maintenance in low/middle schooling	459	44.1	28.3	27.7	-0.6	Ref
	Upward mobility	255	24.5	23.9	25.9	+2.0	0.26
	Maintenance in high schooling	327	31.4	29.9	32.9	+3.0	0.22
Income trajectory							
	Maintenance in 1° or 2° tercile	443	42.6	25.5	26.0	+0.5	Ref
	Upward mobility	199	19.1	27.6	28.1	+0.5	0.01
	Downward mobility	243	23.3	28.8	29.7	+0.9	0.09
	Maintenance in 3° tercile	156	15.0	32.7	36.8	+4.1	0.16
Total		1,041	100.0	27.8	28.9	+1.1	-

Δ variation (%) between 1999 and 2011–12.

Ref reference.

Men who improved their income showed a 0.5% higher increase in vegetable consumption than those who stayed in the first or second tercile of income (p-value=0.01) (Table 2).

Almost half of the women (46.8%) maintained a secondary schooling level, while 20.4% increased education level between 1999 and 2011–12. Over time, there was an overall rise in daily vegetable consumption, growing by 2.4%. By 2012, 44.9% of women reported daily vegetable consumption (Table 3).

**Table 3:** Schooling and income trajectories and daily vegetable consumption according to socioeconomic trajectories among female civil servants at a university in 1999 and 2011–12: *Pró-Saúde* (Pro-Health) Study, Rio de Janeiro, Brazil (n=1,340).

Socioeconomic trajectories		Total		Daily vegetable consumption			
				1999	2012	Δ	p-value
		n	%	%	%	%	
Schooling trajectory							
	Maintenance in low/middle schooling	440	32.8	45.6	48.4	+2.8	Ref
	Upward mobility	273	20.4	41.5	42.9	+1.4	0.14
	Maintenance in high schooling	627	46.8	40.8	43.3	+2.5	0.05
Income trajectory							
	Maintenance in 1° or 2° tercile	464	34.9	41.2	44.8	+3.6	Ref
	Upward mobility	362	26.9	40.1	42.1	+2.0	0.49
	Downward mobility	272	17.9	45.2	39.8	-5.4	0.87
	Maintenance in 3° tercile	242	20.3	45.9	55.0	+9.1	0.02
Total		1,340	100.0	42.5	44.9	2.4	-

Δ variation (%) between 1999 and 2011–12;

Ref reference.

A 2.5% increase in daily vegetable consumption was observed among women who maintained a high schooling level compared to those who remained in low/middle schooling (p-value=0.05) and a 9.1% rise was identified among those who stayed in the higher income category compared to those who continued in the first or second income tertile (p=0.02) (Table 3).

Adjusted models showed that women who reduced their income were less likely to consume vegetables daily after 13 years of follow-up (OR 0.93; 95%CI 0.89–0.97) (Table 4).

**Table 4:** Association between education and income trajectories and daily vegetable consumption among civil servants between 1999 and 2011–12: *Pró-Saúde* (Pro-Health) Study, Rio de Janeiro, Brazil.

Male		Daily vegetable consumption		
		OR	p-value	95%CI
Schooling trajectory				
	Maintenance in low/middle schooling	Reference		
	Upward mobility	0.98	0.23	0.94–1.02
	Maintenance in high schooling	1.02	0.90	0.97–1.03
Income trajectory				
	Maintenance in 1° or 2° tercile	Reference		
	Upward mobility	0.99	0.57	0.95–1.03
	Downward mobility	0.96	0.14	0.92–1.01
	Maintenance in 3° tercile	1.01	0.55	0.98–1.05
Female		Daily vegetable consumption		
		OR	p-value	95%CI
Schooling trajectory				
	Maintenance in low/middle schooling	Reference		
	Upward mobility	1.02	0.39	0.98–1.06
	Maintenance in high schooling	0.99	0.73	0.95–1.03
Income trajectory				
	Maintenance in 1° or 2° tercile	Reference		
	Upward Mobility	0.98	0.28	0.93–1.02
	Downward Mobility	0.93	<0.01	0.89–0.97
	Maintenance in 3° tercile	0.99	0.77	0.96–1.04

OR: odds ratio. Models adjusted by age and schooling or income.

## DISCUSSION

This study found that a decrease in income was associated with a lower likelihood of transitioning to daily vegetable consumption among females after 13 years of follow-up despite the already low prevalence of daily consumption. Moreover, higher-income groups and those experimenting with upward schooling mobility consumed more vegetables than lower-income/schooling groups from a public employee cohort.

Other longitudinal studies found similar results despite using different socioeconomic indicators. A cohort study in the United Kingdom evaluated the association between social mobility and dietary intake, revealing a direct association between stability in higher occupational classes (non-manual workers) and a healthier dietary pattern^13^. In a Finnish cohort, a direct association was observed between stability in professional occupational classes (non-manual workers) and increased consumption of raw vegetables among women^12^.

In a Brazilian birth cohort study, there was an inverse association between socioeconomic status in 2008 and changes in fruit and vegetable consumption between 2008–11. The study showed reduced daily consumption frequency, especially among adolescents from households with the highest socioeconomic level. Another Brazilian cohort study showed that, during the 23-year follow-up period, individuals classified according to change in household income as "never poor" had lower adherence to the Brazilian dietary pattern^24^.

The slight increase in the prevalence of daily consumption of vegetables observed in this study is consistent with data from the Surveillance System of Risk and Protective Factors for Non-Communicable Diseases by Telephone Survey (VIGITEL), which detected a 4.0% increase in the prevalence of recommended fruit and vegetable consumption in Brazilian capitals (from 20 to 24%, between 2008–16), especially among men and young people aged 25–34 years^9^. Moreover, a national study showed that vegetables are still among the 20 most frequently consumed foods in 2018–19 and have experienced a slight increase^15^.

The upward mobility observed in this study in terms of income and education is likely related to various factors, including changes in hiring practices for technical and administrative employees, the implementation of career plans, and the reorganization of promotion criteria that occurred from 2006 onward (State Law No. 6.701/2014 of 12/03/2014). Additionally, the period under investigation coincided with significant economic, social, and demographic transformations in Brazil, which significantly affected the living and working conditions of the population and, in turn, influenced the nutritional transition^29^.

The adjusted analysis shows that the effect of educational variation on consumption does not occur independently of income variation. Household income instability can harm living conditions and reduce the capacity for consumption, particularly among more impoverished populations. Changes in household income or food prices may impact the consumption of healthy foods for those with lower income levels, as their ability to purchase nutritious options is compromised^30^.

A study that assessed the price of food classified per the NOVA system found that natural products and culinary ingredients had a lower price per calorie than other groups, suggesting an economic advantage in preparing meals at home compared to their replacement with ultra-processed foods^21^. While fresh foods (such as meat, milk, fruits, and vegetables) tend to be more expensive than ultra-processed foods, grains (such as rice and beans) emerge as a more economical alternative to adopting healthy eating practices. The prices of vegetables may vary throughout the year due to climatic issues, fuel prices, and other factors, hampering many households’ access to the vegetable group^30^.

Moreover, the modest associations found in our study may be partly attributed to other factors that influence consumption, such as the availability of vegetables in locations outside of the home^31^. Our study population consists of employees who work on the same university campus and are exposed to the same food environment daily^32^. A study conducted with a representative sample of the Brazilian population found that 11–23% of daily fruit and vegetable consumption occurs outside the home^33^. Moreover, vegetable consumption is primarily observed during lunchtime^33^ at snack bars and restaurants^34^, depending on the availability and accessibility of these foods in the visited food environment.

This study has several strengths, including its longitudinal design that enabled the investigation of dietary intake over time. Vegetables have a distinct protective effect against non-communicable diseases (NCDs)^3^. Using equivalent income to investigate income trajectories provides a better weight distribution for each individual in the total cost of family living, making it a robust measure of socioeconomic status. Also, the study appropriately modeled longitudinal data analysis with repeated measures, considering intrapersonal correlation, which was not considered in other studies with similar objectives.

The dietary assessment tool employed in this study had inherent limitations that prevented the evaluation of the recommended vegetable consumption. The reduced food frequency questionnaire did not inquire about the quantity or number of servings consumed.

In conclusion, this study highlights the low prevalence of vegetable consumption and sheds light on the roles of education and income trajectories in shaping the consumption of vegetables in adulthood. The results emphasize the importance of continuous monitoring of disparities in food consumption to identify the most vulnerable population strata and the need to promote structural public policies to increase income and education to reduce the influence of social inequality on vegetable consumption and to expand access to fresh foods. Future research could explore additional determinants of vegetable consumption, including contextual factors, to inform the design of more effective interventions.
